# DNA methylation and gene expression signatures are associated with ataxia-telangiectasia phenotype

**DOI:** 10.1038/s41598-020-64514-2

**Published:** 2020-05-04

**Authors:** Sharon A. McGrath-Morrow, Roland Ndeh, Kathryn A. Helmin, Basil Khuder, Cynthia Rothblum-Oviatt, Joseph M. Collaco, Jennifer Wright, Paul A. Reyfman, Howard M. Lederman, Benjamin D. Singer

**Affiliations:** 1 Eudowood Division of Pediatric Respiratory Sciences, Johns Hopkins School of Medicine, Baltimore, MD USA; 2 Division of Pulmonary and Critical Care Medicine, Northwestern University Feinberg School of Medicine, Chicago, IL USA; 3 A-T Children’s Project, Coconut Creek, FL USA; 4 Eudowood Division of Pediatric, Allergy and Immunology, Johns Hopkins School of Medicine, Baltimore, MD USA; 5 Department of Biochemistry and Molecular Genetics, Northwestern University Feinberg School of Medicine, Chicago, IL USA; 6 Simpson Querrey Center for Epigenetics, Northwestern University Feinberg School of Medicine, Chicago, IL USA

**Keywords:** Translational immunology, Immunological deficiency syndromes, DNA methylation

## Abstract

People with ataxia-telangiectasia (A-T) display phenotypic variability with regard to progression of immunodeficiency, sino-pulmonary disease, and neurologic decline. To determine the association between differential gene expression, epigenetic state, and phenotypic variation among people with A-T, we performed transcriptional and genome-wide DNA methylation profiling in patients with mild and classic A-T progression as well as healthy controls. RNA and genomic DNA were isolated from peripheral blood mononuclear cells for transcriptional and DNA methylation profiling with RNA-sequencing and modified reduced representation bisulfite sequencing, respectively. We identified 555 genes that were differentially expressed among the control, mild A-T, and classic A-T groups. Genome-wide DNA methylation profiling revealed differential promoter methylation in *cis* with 146 of these differentially expressed genes. Functional enrichment analysis identified significant enrichment in immune, growth, and apoptotic pathways among the methylation-regulated genes. Regardless of clinical phenotype, all A-T participants exhibited downregulation of critical genes involved in B cell function (*PAX5*, *CD79A*, *CD22*, and *FCRL1*) and upregulation of several genes associated with senescence and malignancy, including *SERPINE1*. These findings indicate that gene expression differences may be associated with phenotypic variability and suggest that DNA methylation regulates expression of critical immune response genes in people with A-T.

## Introduction

Ataxia-telangiectasia (A-T) is a rare autosomal recessive disease with a prevalence of approximately 1 in 40,000–100,000 live births^1^. The underlying defect in A-T is absence or reduced production of the ATM protein, a phosphoinositide 3-kinase-like enzyme involved in multiple cellular functions including double stranded DNA repair, cell cycle control, tumor surveillance, telomere length monitoring, and apoptosis^2^. Cells with ATM mutations have higher sensitivity to ionizing radiation that results in increased chromosomal breakage, decreased cell survival^3^, and increased malignancy risk^4^.

Infants with A-T are born phenotypically normal. However, most individuals with A-T develop ataxic symptoms as toddlers, often followed by ocular apraxia, telangiectasias, and loss of ambulatory skills in later childhood^5,6^. Many people with classic A-T also have immune defects. These immunodeficiencies most commonly manifest as low or absent IgA levels, low circulating B and T cell numbers, and suboptimal antibody responses to vaccines^7–9^. Nevertheless, a subset of individuals with A-T exhibit functionally normal immune function with milder and slower neurological progression^10^. Despite this contrast in immune profile, individuals with this mild (atypical) A-T phenotype have a higher risk for cancer, similar to the risk for individuals with classic A-T. Indeed, up to 30% of people with A-T will develop cancer during their lifetime^11^. Lymphoid malignancies are most frequent during the first two decades of life, while solid tumors are more common in adults. Adults with A-T also exhibit symptoms of premature aging, including cardiovascular disease, metabolic syndrome, and glucose intolerance^12^.

The majority of people with A-T have compound heterozygous mutations of the *ATM* gene, likely contributing in part to the variability in disease presentation and survival^7,12^. Although some genotype-phenotype correlations have been reported in founder populations, mutational hotspots in the *ATM* gene are not universally found^13–16^. In addition, there are no clinical biomarkers or molecular signatures that predict disease progression, malignancy risk, survival, or response to therapy. Having these tools could help with disease surveillance and improve survival for people with A-T.

Identifying molecular pathways associated with specific A-T phenotypes could also be useful in validating biomarkers. This in turn might help identify subsets of people with A-T who may be at higher or lower risk for developing certain disease-specific conditions such as severe infection and malignancy. Identifying genes and gene processes regulated by DNA methylation could also provide insights into regulatory mechanisms that represent therapeutic targets to modulate gene expression and attenuate disease phenotype in people with A-T^17^. Accordingly, we sought to determine the association between phenotypic variability^10^ and the transcriptional and epigenetic landscapes in people with A-T. We hypothesized that people with mild (atypical) disease progression and people with classic (more severe) disease progression would have different transcriptional and DNA methylation signatures from each other and from non-A-T healthy control study participants. As there are common phenotypic similarities found among all people with A-T, we also hypothesized that participants with A-T regardless of phenotype would share some common gene signatures. Finally, we hypothesized that genes differentially expressed in people with A-T would be regulated in part by DNA methylation.

## Results

*PBMCs from mild and classic A-T study participants display different transcriptomes from one another and from non-A-T controls*. Eight participants with A-T were assigned to mild or classic phenotypes based on a phenotypic classification described by Fievet *et al*.^10^. Participants characterized as mild A-T had ≤ 50% of the standard criteria, whereas participants characterized as classic A-T had > 50% of the standard criteria (*p* < 0.003) (Table 1 and Supplementary Table S1). Controls included four healthy young adults without A-T. We observed a non-statistically significant variability in age between groups (one-way ANOVA, *p* = 0.051; Sidak’s *post hoc* test of non-A-T control group versus classic A-T group, *p* = 0.10). No participants were acutely ill at the time of evaluation. Following grouping of study participants, isolated peripheral blood mononuclear cells (PBMCs) enriched for lymphocytes were used for genome-wide transcriptional and DNA methylation profiling studies with RNA-sequencing (RNA-seq) and modified reduced representation bisulfite sequencing (mRRBS), respectively. We examined complete blood counts obtained from 7/8 A-T participants within less than 1 year of the molecular studies. In participants with A-T, the average lymphocyte percentage was 27.7% (standard deviation, 9.16%; range, 17.0–35.6%) with an average white blood cell count of 9.4 ×10^3^ (standard deviation, 3.18 ×10^3^; range, 4.08 ×10^3^ - 13.8 ×10^3^). These results are consistent with normal or near-normal peripheral lymphocyte counts. To estimate the relative proportion of B and T cells, we applied an *in silico* cellular deconvolution procedure to the bulk RNA-seq data. This approach revealed decreased frequencies of B and T marker gene profiles among study participants with A-T compared with control participants (Supplementary Fig. S1).

**Table 1:** Phenotypic classification of study participants.

Participant	Ataxia (<8 yrs)	Loss of walking (<15 yrs)	Oculomotor ataxia (<15 yrs)	Ocular telangiectasia (<15 yrs)	IgA deficiency	Clinical immuno-deficiency	% Criteria	Type of AT based on % criteria	Age (years)
AT002	Yes	Yes	NK	NK	Yes	No	0.75	Classic	26
AT005	Yes	Yes	Yes	Yes	NK	No	0.80	Classic	29
AT006	Yes	Yes	Yes	Yes	No	No	0.67	Classic	12
AT007	Yes	Yes	Yes	Yes	Yes	No	0.83	Classic	14
AT008	Yes	Yes	Yes	Yes	Yes	No	0.83	Classic	12
AT001	Yes	No	NK	NK	No	No	0.25	Mild	33
AT003	Yes	No	Yes	Yes	No	No	0.50	Mild	29
AT004	Yes	No	NK	Yes	NK	No	0.50	Mild	31
AT009WT	—	—	—	—	—	—	—	WT	21
AT010WT	—	—	—	—	—	—	—	WT	26
AT011WT	—	—	—	—	—	—	—	WT	26
AT012WT	—	—	—	—	—	—	—	WT	31

NK: not known; WT: wild type.

**Figure 1:**
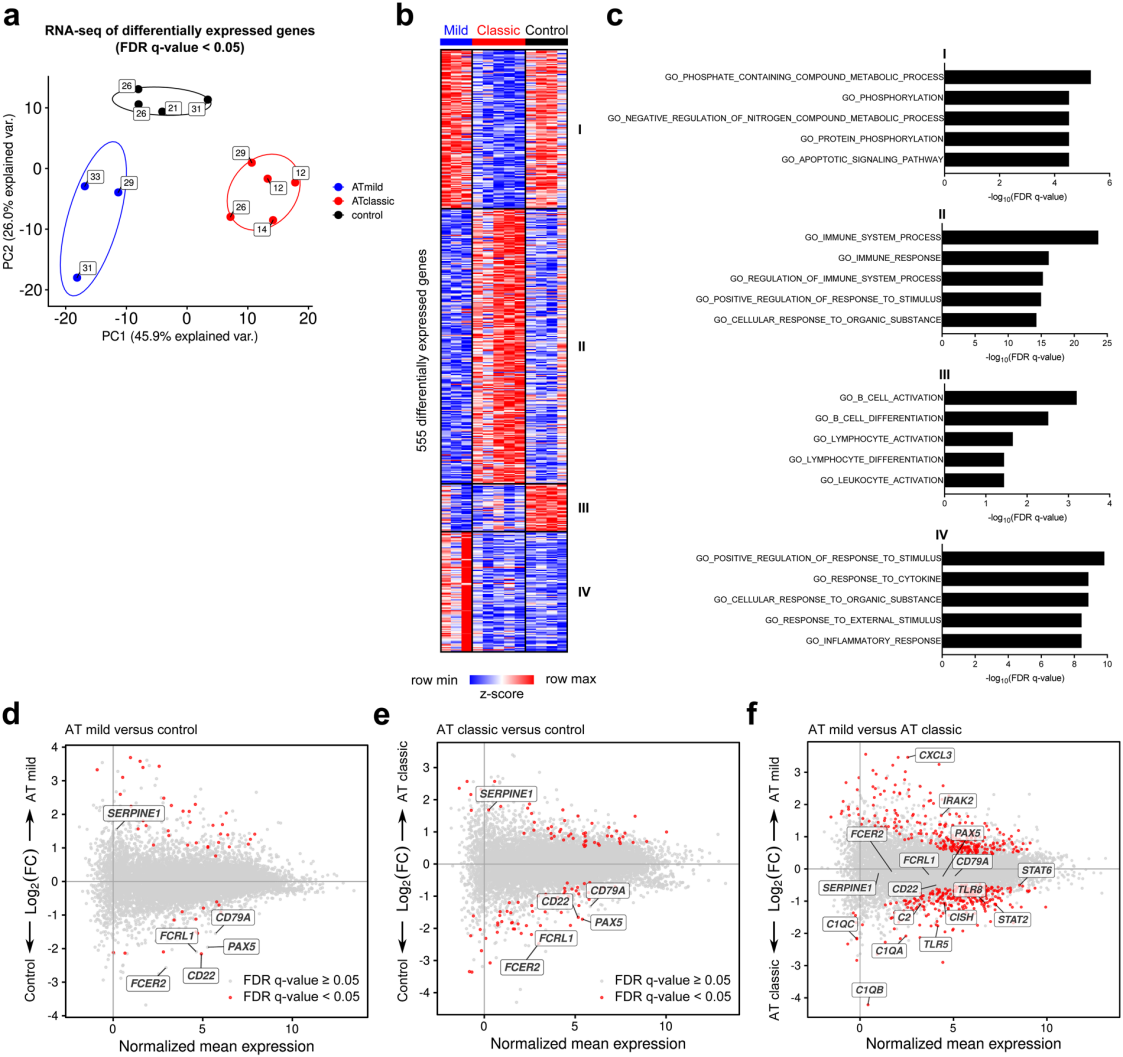
Transcriptional profiling reveals differential gene expression between participants with mild and classic A-T and control study participants. (**a**) Principal component analysis of 555 differentially expressed genes identified from a generalized linear model and ANOVA-like testing with FDR *q*-value <0.05. Ellipses represent normal contour lines with one standard deviation probability. Points are annotated with participant age in years. (**b**) *K*-means clustering of differentially expressed genes with *k* = 4 and scaled as *z*-score across rows. (**c**) Top five gene ontology (GO) processes derived from each *k*-means cluster ranked by -log_10_-transformed false-discovery rate (FDR) *q*-value. (**d-f**) MA plots comparing the gene expression profile of mild A-T versus control participants (d), classic A-T versus control participants (e), and mild A-T versus classic A-T participants (f). Genes of interest are annotated. FC = fold change.

Principal component analysis (PCA) of differentially expressed genes (DEGs) revealed clustering by group assignment (Fig. 1a). PC1 explained the variance between mild and classic A-T phenotypes, and PC2 explained the variance between the A-T and control groups. Multiple group testing with a false-discovery rate (FDR) *q*-value <0.05 revealed 555 DEGs among the three groups, which separated into four *k*-means clusters (Fig. 1b and Supplementary Table S2). *K*-means cluster 1 contained genes down-regulated primarily among A-T participants with a classic phenotype. Gene ontology (GO) processes in cluster 1 included those involved with apoptosis and regulation of phosphate and nitrogen reactions (Fig. 1c). Gene ontology (GO) processes associated with *k*-means cluster 2 were mainly upregulated in the classic A-T participants and included immune system processes. *K*-means cluster 3 included GO processes involved with lymphocyte development and B cell morphology and function. These processes were downregulated in both mild and classic A-T participants compared with control participants. In *k*-means cluster 4, GO processes involved with rate of response to stimulus and cell activity were primarily upregulated in A-T participants with mild phenotypes. Pairwise comparisons revealed transcriptional differences between A-T groups and control participants as well as between mild and classic A-T phenotypes (Fig. 1d–f).

**Figure 2:**
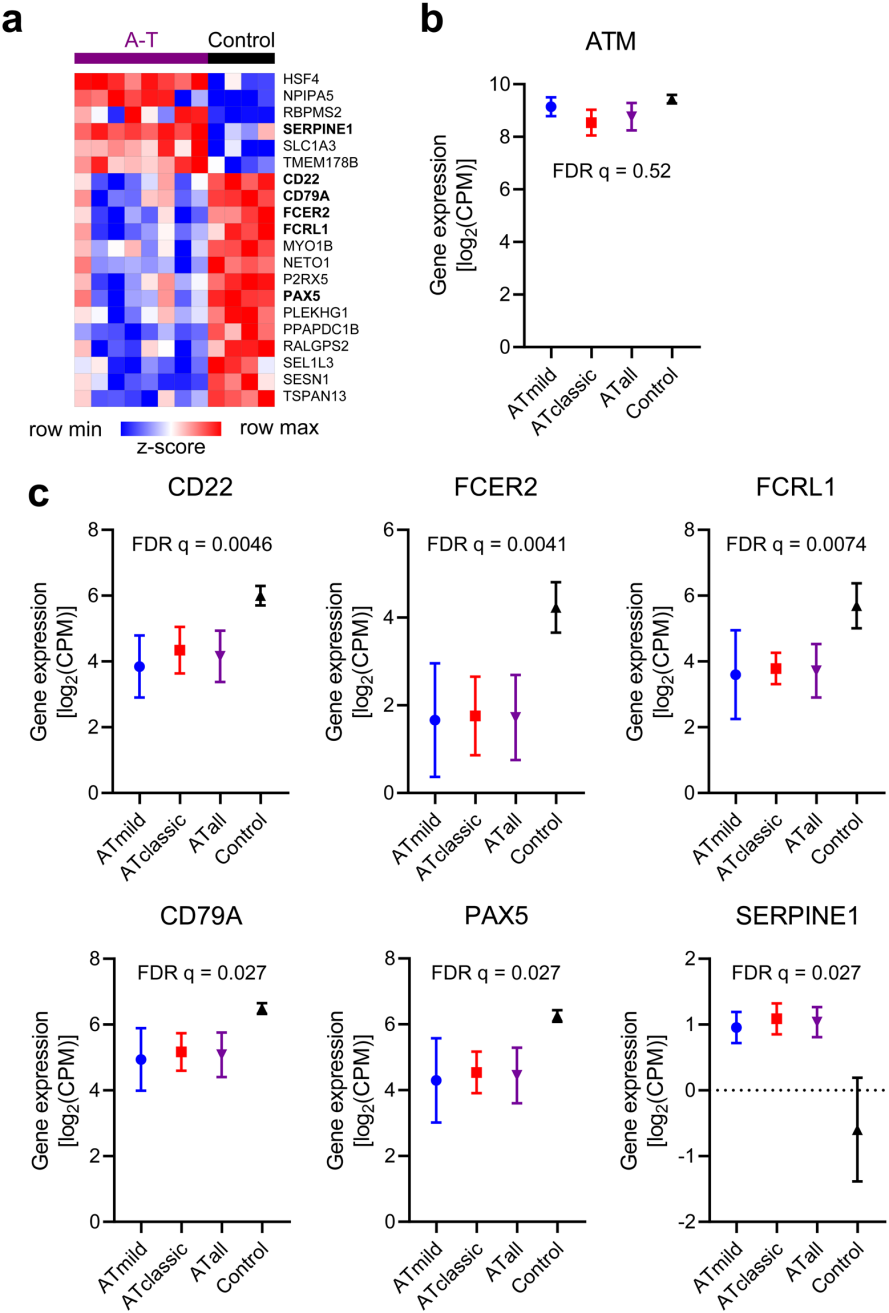
A shared transcriptomic signature distinguishes participants with A-T from control participants. (**a**) *K*-means clustering of 20 differentially expressed genes with *k* = 2 and scaled as *z*-score across rows. Selected genes are highlighted. (**b**) Gene expression of *ATM* in mild A-T, classic A-T, all A-T, and control participants. (**c**) Gene expression of selected genes in mild A-T, classic A-T, all A-T, and control participants. Plots show mean ± standard deviation. FDR *q*-values for the pairwise test comparing the merged A-T group with non-A-T controls is shown for (b and c). CPM = counts per million.

*A core set of transcriptional responses is common to both mild and classic A-T participants compared with control participants*. We recognized similarities among the mild and classic A-T signatures and identified a core set of 20 DEGs expressed in both mild and classic A-T participants when compared with control participants (Fig. 2a and Supplementary Table S3). *ATM* expression was not significantly different comparing the mild A-T group with non-A-T controls (FDR *q*-value ≈ 0.9); however, the classic A-T group exhibited lower *ATM* expression compared with non-A-T controls (FDR *q*-value ≈ 0.05). Thus, when the A-T groups were merged together, *ATM* expression was not significantly different from non-A-T controls (Fig. 2b). DEGs downregulated in both mild and classic A-T participants included *PAX5* and *FCRL1* (involved in B cell development), *FCER2* (involved in IgE synthesis), *CD22* (inhibitor of B cell responses), and *CD79A* (involved in transduction of B cell signals) (Fig. 2c)^18–23^.

Of additional interest was the finding that *SERPINE1* was upregulated in all A-T participants regardless of phenotype (Figs. 1d,e and 2c). *SERPINE1* encodes plasminogen activator inhibitor-1 (PAI-1), a protein elevated in conditions of premature aging, insulin resistance, and coronary heart disease^24^. Other genes upregulated among A-T participants included *HSF4*, *NPIPA5*, *RBPMS2*, *SLC1A3*, and *TMEM178B*. Functions of these genes include regulation of heat shock proteins, stimulation of visceral smooth muscle proliferation, and episodic ataxia with gain-of-function mutations^25–27^. The shared DEGs identified among participants with A-T included genes linked to conditions well described in patients with A-T, including early senescence, the presence of ataxia, the development of malignancy, B cell immune dysregulation, and metabolic syndrome^28^.

**Figure 3:**
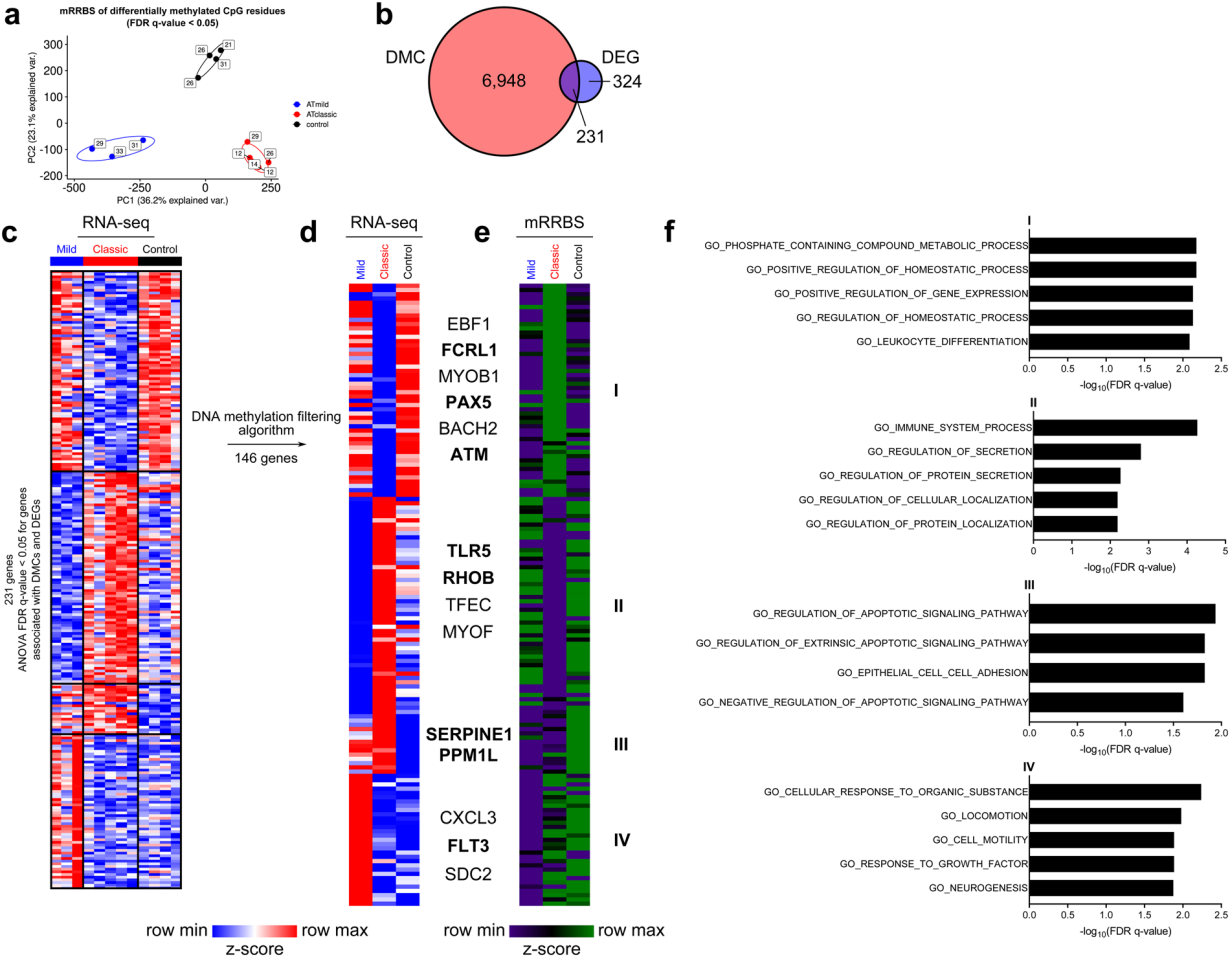
Distinct DNA methylation profiles identify A-T phenotypes. (**a**) Principal component analysis of 66,410 differentially methylated CpGs (DMCs) identified from a beta-binomial regression model with an arcsine link function fitted using the generalized least square method and Wald-test FDR *q*-value <0.05. Ellipses represent normal contour lines with one standard deviation probability. Points are annotated with participant age in years. (**b**) Venn diagram partitioning genes associated with differentially methylated cytosines (DMCs) within 2 kb of their gene bodies (inclusive) and differentially expressed genes (DEGs), all with FDR *q*-value <0.05. (**c**) *K*-means clustering of 231 DEGs associated with differentially methylated CpGs (DMCs); *k* = 4 and scaled as *z*-score across rows. (**d**) Results of filtering promoter (transcriptional start site ± 1 kb) CpGs by 25% difference in CpG methylation between groups. For each *k*-means cluster, CpGs with 25% higher methylation in lower expression groups compared with higher expression groups passed the filter. The heat map shows average gene expression (log_2_-transformed read counts per million) for 146 genes passing the methylation difference filter scaled as *z*-score across rows. (**e**) Merged-replicate average CpG methylation in gene promoters scaled as *z*-score across rows for the 146 loci passing the methylation difference filter. For (d) and (e), genes are ordered as in (c). (**f**) Top five gene ontology (GO) processes derived from each *k*-means cluster ranked by -log_10_-transformed FDR *q*-value for the genes in (e and f).

*DNA methylation is statistically likely to regulate gene expression differences in A-T*. To determine whether DNA methylation is associated with differential gene expression among these groups, we performed genome-wide DNA methylation profiling using modified reduced representation bisulfite sequencing (mRRBS)^17,29^. PCA revealed differentially methylated cytosine-phospho-guanine residues (CpGs) between mild A-T, classic A-T, and non-A-T controls (Fig. 3a) that resembled the PCA of DEGs shown in Fig. 1a. We identified 231 genes that were both differentially expressed in the RNA-seq data set and whose promoters were differentially methylated in the mRRBS data set (Fig. 3b). We then applied a previously-published DNA methylation difference-filtering algorithm designed to identify methylation-regulated genes within this group^29,30^. This procedure identified 146 genes (Fig. 3c–e and Supplementary Table S4) including *FCRL1*, *PAX5*, *SERPINE1*, *PPM1L*, *TLR5*, *RHOB*, and *FLT3*. *K*-means cluster 1 genes were enriched for GO processes involved in homeostatic and regulatory processes (Fig. 3f). GO processes in *k*-means cluster 2 included immune system and regulatory processes involved with protein secretion and localization. *K*-means cluster 3 included processes involved in apoptotic signaling, and *k*-means cluster 4 included processes involved in cell motility and growth. A network analysis of the gene body loci for these 146 genes highlighted immune dysregulation (Supplementary Fig. S2). Collectively, these data sets reveal methylation-regulated gene expression differences both between phenotypes of A-T and between A-T and control participants.

## Discussion

In this study, we found that variability in A-T phenotype correlated with changes in gene expression and epigenetic signature. These findings suggest that differences in transcriptional programs may underlie the phenotypic variability observed in people with mild and classic A-T. We also found a core set of DEGs that were shared by both mild and classic phenotypic participants but differed from non-A-T controls. These shared DEGs included genes involved in B cell development, senescence, and tumor suppression—processes known to be dysregulated among people with A-T^1^. Additionally, we found an association between *cis* promoter site methylation and gene expression in a subset of immune response genes, indicating that epigenetic mechanisms may regulate gene expression of critical immune response genes among people with A-T.

Transcriptional profiling revealed distinct expression signatures between mild and classic A-T phenotypes. This separation between groups accounted for the plurality of variance observed in the principal component analysis and indicated that mild and classic A-T participants were transcriptionally different from each other.

ATM is part of the DNA damage response and maintains genomic integrity by responding to DNA damage through p53-induced cell cycle arrest, repair, and apoptosis. It has previously been shown that apoptosis is activated through ATM via miR-34a/HDAC1^31,32^. ATM is a regulator of DNA-PKcs, which binds to DNA ends and senses breakage^33^. In addition to the ATM pathway, DNA-PKcs can signal through other pathways including c-ABL, p21, and Tap73, which can initiate apoptosis through PUMA, NOXA, and BAX. Thus, apoptosis would be expected to be dysregulated in ATM-deficient individuals, since the overall DNA damage response is altered. In our study, when including all DEGs, participants with classic A-T had lower functional enrichment in apoptotic signaling pathways compared with mild A-T and non-A-T controls. However, when examining only DEGs associated with differentially methylated cytosines (DMCs), we found increased enrichment in apoptotic processes in mild and classic A-T participants compared with non-A-T controls. The participants with classic A-T phenotypes also displayed enrichment of immune processes compared with mild A-T and non-A-T controls. DEGs upregulated in classic A-T participants included the immune response genes *TLR5*, *TLR8*, *STAT2*, and *STAT6* as well as genes encoding complement components (*C1QA*, *C1QB*, *C1QC*, and *C2*). *CISH*, a gene encoding protein that negatively regulates cytokine signaling^34^, was also upregulated among classic A-T participants. These findings suggest that people with a classic A-T phenotype exhibit an upregulated immune response at baseline whereas those with mild A-T phenotypes do not. This dysregulated immune program may be compensatory for the ineffective host response observed among people with classic A-T.

Although ATM protein levels were not measured in this study, *ATM* gene expression was higher in mild A-T participants compared with those with classic A-T. The participants with mild A-T also had higher expression of genes involved in inflammation, including *CXCL3* and *IRAK2*, and GO processes associated with upregulation of cell migration and cell growth. These inflammatory and growth signatures found in participants with mild A-T but not classic A-T could signify an adaptive response to DNA injury unique to people with mild A-T^35,36^. This inflammatory transcriptional signature in the mild A-T participants could also contribute to the higher malignancy risk reported among people with A-T and carriers of *ATM* mutations^37–40^.

We identified a core set of differential transcriptional responses present in both mild and classic A-T participants. *PAX5*, *CD79A*, *CD22*, and *FCRL1* are members of a core set of genes that are critical for B cell development and function^41^. These genes were downregulated among all participants with A-T compared with non-A-T controls. Furthermore, in addition to the role of *PAX5* in B cell development, this gene also acts as a tumor suppressor^22,42^. Indeed, downregulation and dysregulation of *PAX5* may contribute to the higher risk of malignancy, particularly B cell lymphomas, observed among people with A-T^43,44^. We also found that *SERPINE1* was upregulated among all participants with A-T compared with non-A-T controls. *SERPINE1* has been shown to mediate cellular senescence, and a null mutation in *SERPINE1* has been associated with longer telomere length, lower prevalence of diabetes, and longer life span in an extended Amish kindred with a null mutation^24^. Supporting a possible link between elevated *SERPINE1* and A-T phenotype are the observations that people with A-T have accelerated telomere shortening^45^, and that premature aging is increasingly recognized in A-T as overall life expectancies have increased with effective supportive care^12^.

Using a previously published computational tool^29,30^, we found that *cis* DNA methylation was associated with gene expression at the loci of 146 genes that are predominantly involved with immune responses. This finding indicates that DNA methylation differs between participants with mild and classic A-T phenotypes and non-A-T controls and provides insights into the mechanisms that regulate gene expression in A-T. Recognition of DNA methylation as a regulator of gene expression may offer opportunities to modulate abnormal gene expression among people with A-T. These findings also raise mechanistic questions regarding the link between DNA damage and differential methylation patterning that are beyond the scope of our study.

As A-T is a rare disease, limitations to our study include the small number of participants in each group. Nevertheless, the differences in transcriptional signatures between the two A-T groups and non-A-T controls were striking. While some DEGs were similar between A-T phenotypes, many of the DEGs and cellular processes diverged between the two A-T phenotypes, consistent with the observed variability in clinical phenotypes present in people with A-T. The non-A-T controls in this study were older than the classic A-T participants. Although the difference in age was not statistically significant, age could represent a potential confounder in this study. We used an isolation approach to enrich for lymphocytes. Nevertheless, this approach would not necessarily normalize frequencies of B and T cell subsets between groups; indeed, our deconvolution procedure revealed a decreased proportion of B and T cell marker gene profiles comparing the A-T groups with the control group. These results can be interpreted to mean a decrease in the representation of these cell types in the cells subjected to bulk RNA-seq. Alternatively, as the analysis relies on cell type-specifying marker genes, these results may also represent a decrease in expression of these genes in otherwise balanced cell populations. Regardless, in this study each sample consisted of a composite of lymphocyte populations, representative of the group. Additionally, the use of bulk RNA-seq on mixed cell populations allowed for aggregate measures to reflect the distinct milieus of mild and classic A-T and to include rare populations of cells. Future studies examining molecular signatures in specific lymphocyte subsets may provide a more granular perspective into the role of ATM in immune regulation.

In summary our findings indicate that gene expression differences are associated with phenotypic variability and that DNA methylation may regulate the transcriptional expression of several critical immune response genes among people with A-T.

## Methods

### Participants

The work described has been carried out in accordance with The Code of Ethics of the World Medical Association (Declaration of Helsinki) for studies involving humans. The institutional review board of the Johns Hopkins Medical Institutions approved the study (IRB NA_00051764), and written informed consent was obtained from every participant and/or his/her guardian. All participants with A-T met the diagnostic criteria for A-T based on clinical symptoms and laboratory findings of either elevated alpha-fetoprotein, diminished ATM protein, pathogenic *ATM* mutations, and/or increased chromosomal breakage after *in vitro* exposure to x-rays, as previously established^46^. Demographic information was obtained from chart review. Participants were recruited sequentially from the outpatient Johns Hopkins A-T Clinic and assigned to either a mild or classic A-T phenotype based on a phenotypic classification previously described by Fievet *et al*.^10^. Healthy non-A-T young adults were recruited as controls.

### Isolation of peripheral blood mononuclear cells (PBMCs)

PBMCs were isolated using the Ficoll-Paque Plus Method (GE Healthcare Bio-sciences AB) using a process that maintains the viability of B and T lymphocytes to account for relative lymphopenia among A-T versus control participants as previously described^47^. Briefly, a 7-mL venous blood sample was transferred into a 50-mL conical tube and PBS was added to achieve a 20-mL volume. 15 mL of Ficoll-Paque Plus was transferred into a separate 50-mL conical tube using a syringe. The 20 mL of diluted blood was gently layered onto the Ficoll. The sample was spun at 400 ×*g* for 30 minutes at room temperature with no brake. The PBMC layer was then collected at the diluted plasma/Ficoll interface. PBS was added to the PBMCs to bring the volume up to 20 mL. The sample was then spun at 200 ×*g* for 10 minutes at room temperature. The supernatant was discarded and the cells were resuspended in 1 mL of PBS and counted using a TC20 automatic cell counter (Bio-Rad). 4 mL of PBS was added to the sample and spun at 200 ×*g* for 10 minutes to remove any contaminating Ficoll, platelets, and plasma proteins. For transcriptional and DNA methylation profiling, the sample was resuspended in 350 µL of RLT plus (Qiagen) containing 1% beta-mercaptoethanol, vortexed for 30 seconds, and then transferred to a −80 °C freezer.

### RNA sequencing and analysis

RNA was isolated using the Qiagen AllPrep DNA/RNA Micro kit. Transcriptional profiling was performed as previously described^30,47–49^ with the following modifications. Libraries for RNA sequencing were prepared with the NEBNext Ultra I RNA Library Prep Kit for Illumina with poly(A) mRNA selection (12 ng input) and sequenced using 1 ×75 single-end reads on an Illumina NextSeq 500 instrument using a NextSeq 500/550 V2 High Output reagent kit. Fastq files were trimmed using Trimmomatic v0.38 to remove end nucleotides with a phred score less than 30 while requiring a minimum length of 20 bp and then aligned to the hg38 reference genome using Tophat v.2.1.0 with default parameters. Code used for RNA-seq data processing is available from https://github.com/ebartom/NGSbartom. The RNA-seq quantitation pipeline in SeqMonk v1.44.0 was used to generate gene and transcript expression levels for protein-coding genes (counts data for uniquely mapped reads over exons). Lowly expressed genes were excluded by including only those genes with more than 1 count per million in at least 3 of the samples. Library sizes were re-computed after filtering these genes, and sample normalization factors were calculated and applied using the trimmed mean of M values (TMM) procedure as implemented in edgeR v3.24.3 (R v3.5.1 with RStudio v1.2.1578). ANOVA-like testing and pairwise comparisons (either treating A-T participants by phenotype [Fig. 1] or merging all A-T participants [Fig. 2], respectively) were performed by fitting a generalized linear model and executing a likelihood ratio test followed by false-discovery rate (FDR) correction using the Benjamini-Hochberg method in edgeR^50^. An FDR *q*-value cutoff of 0.05 was used to identify differentially expressed genes. Functional enrichment analysis for Gene Ontology (GO) biologic processes was performed using Molecular Signatures Database (MSigDB) (The Broad Institute).

Bulk RNA-seq deconvolution was performed using the 10X Genomics PBMC 3 K data set as a single-cell RNA-seq reference (https://support.10xgenomics.com/single-cell-gene-expression/datasets/1.1.0/pbmc3k). The PBMC reference had been previously processed and clustered into seven distinct cell-types using Seurat^51^. Reference counts were library-size corrected and log-transformed with SCANPY^52^, and 2,000 highly variable genes were identified and used for downstream analysis. AutoGeneS selected 600 marker genes through 5,000 generations of its optimizer that minimized correlation and maximized Euclidean distance amongst the gene-set^53^. These marker genes were then used to estimate cell type proportions within the bulk RNA-seq data set. A Mann-Whitney U test was used to test significance between the merged A-T and control groups.

### Modified reduced representation bisulfite sequencing and analysis

Genome-wide DNA methylation profiling was performed using modified reduced representation bisulfite sequencing (mRRBS) as previously described^29,30,48,49,54,55^. “Modified” refers to bisulfite conversion of MspI-digested and size-selected DNA prior to random priming, adapter ligation, and indexing; see Singer (2019) for detailed explanation^29^. 250–300 ng of genomic DNA was used as input. Code for mRRBS data processing can be found in the supplement to Singer (2019)^29^. Bisulfite conversion efficiency averaged 99.6% (standard deviation, 0.05%) as estimated by the measured percent of unmethylated CpGs in λ-bacteriophage DNA (New England BioLabs N3013S) added at a 1:200 mass ratio to each sample. In total, we determined the methylation status of 6,697,115 unique CpGs (nearly 25% of the CpG methylome). Beta scores at individual CpG positions were quantified using the SeqMonk platform with the bisulphite feature methylation pipeline. Bismark coverage (counts) files for cytosines in CpG context were analyzed with respect to differential methylation with the DSS v2.34.0 R/Bioconductor package. To detect differentially methylated CpGs, we used DSS to generate a beta-binomial regression model with an arcsine link function fitted using the generalized least square method and then applied a Wald-test with FDR *q*-value <0.05. Putative differentially methylated genes were identified as those containing a differentially methylated cytosine in their promoter, defined as the transcriptional start site ± 1 kb. Our previously described methylation difference-filtering algorithm then selected for promoter CpGs with a methylation directionality (hypermethylated or hypomethylated) inverse to the corresponding gene expression directionality (down- or up-regulated) between groups^29,30^. We used a threshold of 25% for the difference-filtering procedure to establish the list of 146 methylation-regulated genes. Using the gene body coordinates of the 146 methylation-regulated genes, we performed network analysis via the GREAT tool^56^ with the basal+extension association rule (constitutive 5 kb upstream and 1 kb downstream, up to 1000 kb max extension) against the whole genome as background.

### Statistical analysis

Principal component analysis was performed with the prcomp base R statistical function. *K*-means clustering and heat maps were generated using the Morpheus web interface (https://software.broadinstitute.org/morpheus/). *K* was selected using the elbow method. Venn diagrams were created with the VennDiagram v1.6.20 R package. All indicated tests were two tailed unless otherwise stated. Computational analysis was performed using “Genomics Nodes” and “Analytics Nodes” on Quest, Northwestern University’s High-Performance Computing Cluster.

## Supplementary information

Supplementary Information.

## Data Availability

The raw processed sequencing data set (counts and coverage tables) is available from the GEO database under accession number GSE142844 (https://www.ncbi.nlm.nih.gov/geo/). For other original data, please contact the corresponding author.
