# Supplementary material for: DNA methylation and gene expression signatures are associated with ataxia-telangiectasia phenotype
Source: Sci Rep. 2020 May 4;10:7479. doi: 10.1038/s41598-020-64514-2 (PMC7198504; doi:10.1038/s41598-020-64514-2)
Supplement: Supplementary file 1 — Supplementary Information. [file 41598_2020_64514_MOESM1_ESM.pdf]

Supplement

DNA methylation and gene expression signatures are associated with ataxia-telangiectasia phenotype

Sharon A. McGrath-Morrow[1,*], Roland Ndeh[1], Kathryn A. Helmin[2], Basil Khuder[2], Cynthia Rothblum-Oviatt[3], Joseph M. Collaco[1], Jennifer Wright[4], Paul A. Reyfman[2], Howard M. Lederman[4], Benjamin D. Singer[2,5,6]

[1]Eudowood Division of Pediatric Respiratory Sciences, Johns Hopkins School of Medicine, Baltimore, MD

[2]Division of Pulmonary and Critical Care Medicine, Northwestern University Feinberg School of Medicine, Chicago, IL

[3]A-T Children's Project, Coconut Creek, FL

[4]Eudowood Division of Pediatric, Allergy and Immunology, Johns Hopkins School of Medicine, Baltimore, MD

[5]Department of Biochemistry and Molecular Genetics, Northwestern University Feinberg School of Medicine, Chicago, IL

[6]Simpson Querrey Center for Epigenetics, Northwestern University Feinberg School of Medicine, Chicago, IL

***Correspondence:** Dr. Sharon McGrath-Morrow, Eudowood Division of Pediatric Respiratory Sciences, David M. Rubenstein Building, Suite 3075B, 200 North Wolfe Street, Baltimore, MD 21287-2533. Telephone # (410) 955-2035; Fax # (410) 955-1030. E-mail address: smcgrath@jhmi.edu

Supplementary figures

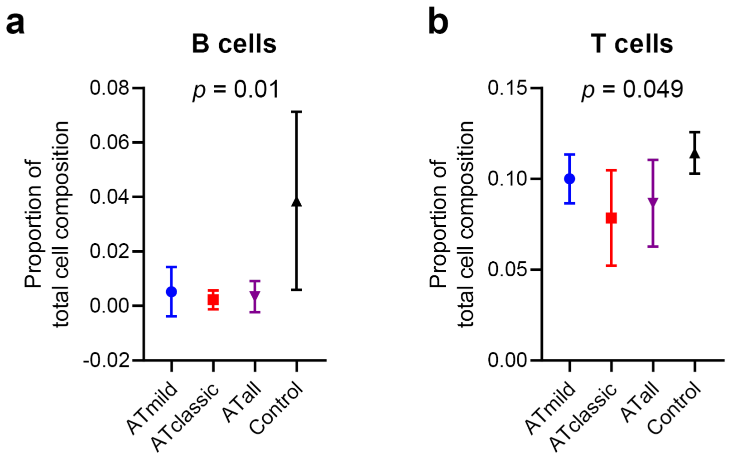

Supplementary Figure S1. Cellular deconvolution analysis of RNA-seq data. (**a**) B cells and (**b**) T cells are shown as a proportion (frequency) of the total cellular composition. The *p*-value (exact) resulting from a Mann-Whitney test comparing the merged A-T group with non-A-T controls is shown.

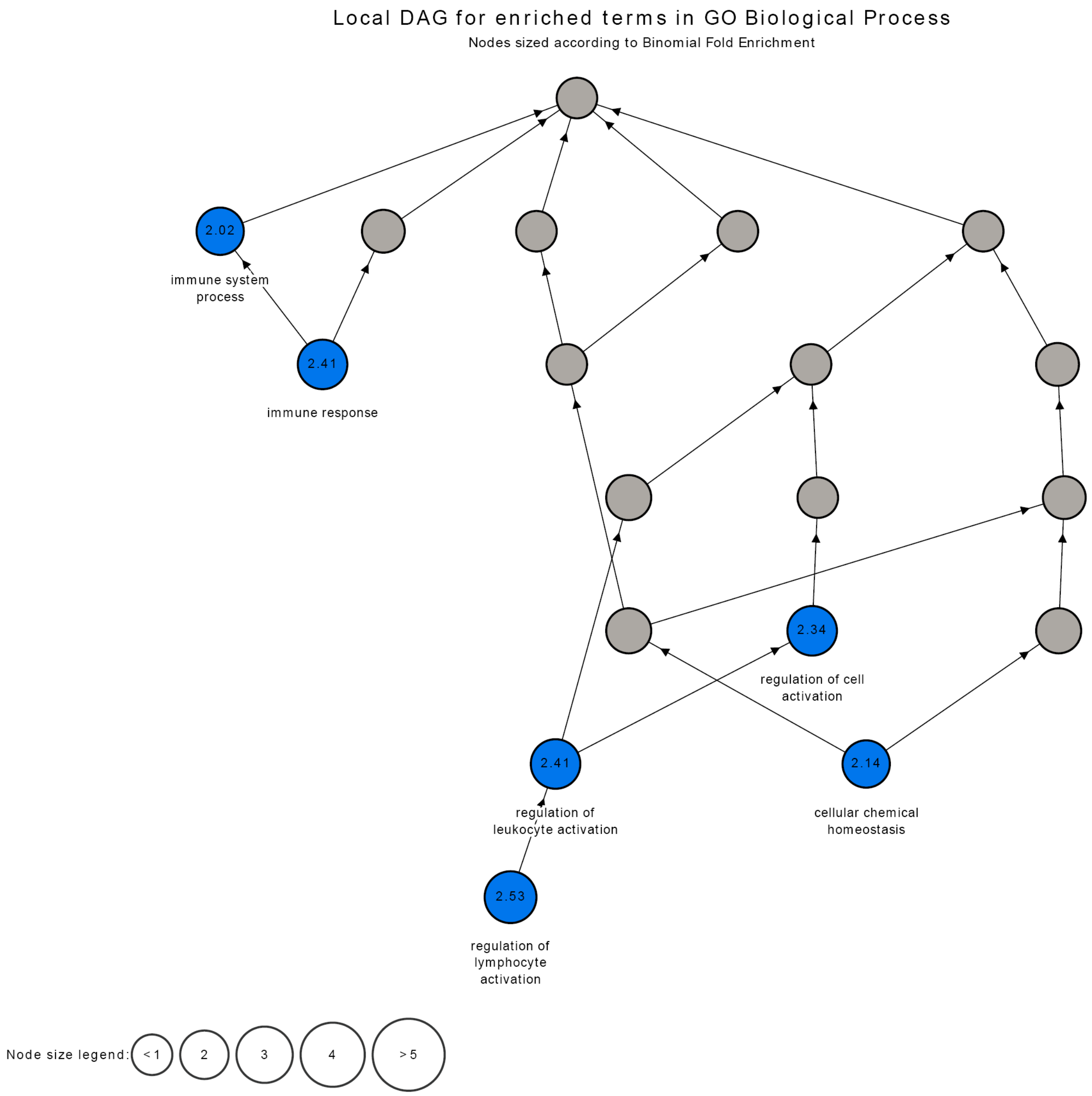

Supplementary Figure S2. Network analysis of methylation-regulated gene loci reveals immune dysregulation. Directed acyclic graph (DAG) based on the enriched terms. Nodes are sized according to binomial fold enrichment.

Supplementary tables

Supplementary Table S1. Extended phenotypic data of study participants. FVC = forced vital capacity.

Participant	Type of AT based on % criteria	Alpha fetoprotein level (ng/ml)	ATM mutation 1	ATM mutation 2	FVC (%)	Malignancy	Fatty liver
**AT002**	Classic	106	c.1027_1030del	Deletions of exons 62-63	57% (2.52l)	*yes	yes
**AT005**	Classic	373	NK	NK	88% (3.93 l)	no	yes
**AT006**	Classic	200	c.2921+1G>A	NK	67% (1.15l)	*yes	no
**AT007**	Classic	252	c.217_218delGA	c.7792C>T	52% (1.85l)	no	no
**AT008**	Classic	90	c.170G>A	c.8792G>A	58% (1.36l)	no	no
**AT001**	Mild	36.8	c.7638_7646delTAGAATTTC	c.7328G>A	94% (3.37l)	no	no
**AT003**	Mild	14	NK	NK	94% (3.34l)	no	yes
**AT004**	Mild	NK	IVS59+1del4	NK	62% (2.53l)	no	yes

*Malignancy diagnosed after clinic visit

Supplementary Table S2. Genes, positions, absolute/relative expression levels, and statistical analysis for genes displayed in Fig. 1b. The table is ordered as Fig. 1b. Absolute expression values for each sample are shown as $\log_2$-transformed read counts per million. Relative expression values are shown as $\log_2$-transformed fold-change compared with the ATclassic group. The column labeled as k.cluster denotes k-means cluster assignment from Fig. 1b. [provided as a tab-delimited file]

Supplementary Table S3. Genes, positions, absolute/relative expression levels, and statistical analysis for genes displayed in Fig. 2a. The table is ordered as Fig. 2a. Absolute expression values for each sample are shown as $\log_2$-transformed read counts per million. Relative expression values are shown as $\log_2$-transformed fold-change compared with the control group. The column labeled as k.cluster denotes k-means cluster assignment from Fig. 2a. [provided as a tab-delimited file]

Supplementary Table S4. List of genes displayed in Fig. 3, d and e. The table is ordered as Fig. 3, d and e. The column labeled as k.cluster denotes k-means cluster assignment from Fig. 3c. [provided as a tab-delimited file]
